# “Do I buy my children shoes, or do I get a compression garment for my lymphoedema?” Australian stakeholder perspectives on cancer-related lymphoedema care

**DOI:** 10.1007/s11764-025-01770-z

**Published:** 2025-03-13

**Authors:** Bogda Koczwara, Jane Lee, Navaz Naghavi, Monique Bareham, Matthew P. Wallen, Neil Piller, Raymond Javan Chan

**Affiliations:** 1 Flinders Medical Centre, Bedford Park, Australia; 2 Flinders University, Bedford Park, Australia; 3 South Australia Health Lymphoedema Compression Garment Advisory Group, Adelaide, Australia

**Keywords:** Cancer-related lymphoedema, Lymphoedema management, Consumer-centred healthcare

## Abstract

**Purpose:**

To identify the experiences and challenges some cancer survivors face in managing lymphoedema and to explore how they and healthcare professionals can best address them.

**Methods:**

A qualitative participatory methodology was employed, involving two stakeholder consultation workshops (one face-to-face and one online). Sessions were audio-recorded, and thematically analysed. Workshops involved 55 participants, comprising people with lived experience of diverse cancers (*n* = 19) and multidisciplinary healthcare professionals (*n* = 36) in Australia.

**Results:**

Participants identified three key challenges: awareness, access, and financial burden. These informed the identification of three key enablers for better consumer-centred care: patient and provider education/training, care pathway, and recognition of lymphoedema as a chronic disease. The need for a national registry system was identified as a key enabler to quantify the burden of disease to support equitable access to resources and treatment.

**Conclusion:**

Cancer survivors at risk of, or experiencing lymphoedema, face significant challenges that could be overcome through initiatives prioritising self-management education and clinician training, navigation, and reimbursement for care.

**Implications for Cancer Survivors:**

Lymphoedema risk reduction and management remains a neglected aspect of survivorship care but survivors and healthcare providers identify a number of strategies to improve lymphoedema care that warrant examination.

## Introduction

Cancer-related lymphoedema (CRL), a chronic swelling of a limb caused by dysfunctional lymphatics as a result of cancer or its treatment, is one of the most distressing consequences of cancer treatment, associated with significant personal and societal cost [1, 2]. CRL affects approximately 20% of cancer survivors, particularly those with breast, genitourinary, gynaecological, or melanoma cancers. Incidence rates vary by type, with lymphoedema affecting 36–47% survivors of vulval cancer, 24% of cervix cancer, 20% of survivors of breast cancer, and 9–29% of melanoma survivors [3, 4].

Lymphoedema involves abnormal lymphatic fluid accumulation, causing swelling, pain, and restricted mobility, requiring long-term management [5, 6]. Those at risk of CRL require lifelong risk reduction strategies, management, and surveillance. Despite its prevalence and significant impact on cancer survivors’ quality of life, CRL often remains under-recognised and inadequately managed [7]. The initial symptoms of lymphoedema, such as numbness, heaviness, and soreness, are often overlooked, resulting in delayed diagnosis and treatment [8].

Effective lymphoedema management requires a holistic, patient-centred approach that includes risk reduction, early detection, and continuous care [9]. In Australia, gaps in lymphoedema care are evident, largely due to an imbalance between service availability and consumer demand, with a shortage of specialised practitioners, particularly in rural and remote areas and limited awareness and expertise in lymphoedema management of general practitioners, nurses, and non-lymphoedema specialist allied health providers [10]. Limited service availability makes early diagnosis and intervention challenging, increasing the demand for more intense care as the condition progresses [11–13].

To understand the experiences and priorities for care improvement, we explored the perspectives of diverse stakeholders involved in lymphoedema care, including cancer survivors, healthcare professionals, lymphoedema researchers, and representatives from support organisations about their views how best to deliver care within the Australian healthcare system.

## Methods

### Study design

A qualitative research design was employed using two rounds of stakeholder consultation workshops to gather diverse perspectives on the challenges and enablers in lymphoedema care. This approach aligns with the exploratory nature of the study, which aimed to capture the insights of individuals with or at risk of CRL, as well as healthcare professionals who provide their care and support.

### Selection of participants

Participants were recruited via purposive sampling through professional networks of authors, including oncology, lymphoedema advocacy, and healthcare communities, to ensure a diverse and relevant participant pool. The stakeholders in this study consisted of two main groups. The first group included consumers, who we identified as individuals living with and beyond cancer who have firsthand experience with lymphoedema challenges or are at risk of developing lymphoedema. The second group comprised healthcare professionals from various health disciplines, including general practitioners, lymphoedema therapists (physiotherapists or occupational therapists, nurses, massage therapists), researchers specialising in lymphoedema, and representatives from lymphoedema support organisations. As this category of participants were all involved in providing care or support to consumers, they are collectively referred to as healthcare professionals throughout the study.

A total of 95 potential participants from the authors’ network were approached via email and 55 provided informed consent to participate in the study (57.8% response rate). Of these, 24 attended the in-person session in Adelaide, South Australia, while 31 joined online from across Australia, with the highest representation from New South Wales (*n* = 8, 14.5%). Among the 55 participants, 18 (32.7%) reported residing in regional and rural areas. The participant group included 19 consumers and 36 healthcare professionals. Among the consumer participants, 15 (78.9%) had breast cancer, 2 (10.5%) had gynaecological cancer, and 2 (10.5%) had melanoma.

**Fig. 1:**
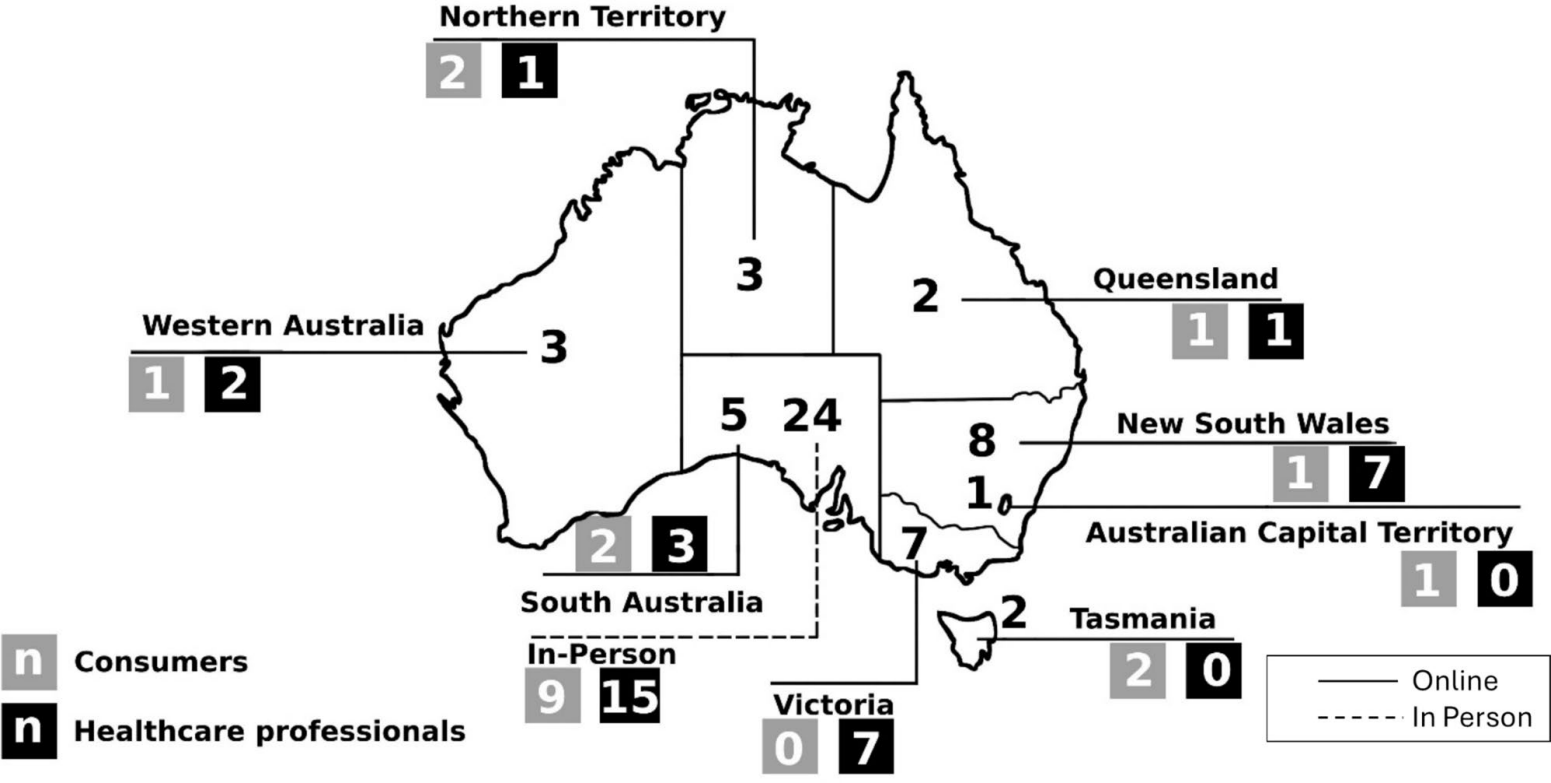
Geographical distribution of participants for in-person and online sessions

Figure 1 illustrates the geographical distribution of participants across Australian states and territories for both in-person and online workshops.

### Study procedures

Both sessions were facilitated by Author 1 (BK), an experienced medical oncologist with expertise in cancer survivorship and qualitative research methodology, and Author 4 (MB), a breast cancer survivor and lymphoedema advocate.

The discussions were structured around several key themes, including participants’ experiences managing lymphoedema, the adequacy of education and awareness, referral pathways, financial burdens, and proposed solutions. The facilitators used open-ended questions to encourage participants to share their personal experiences and perspectives. Probing questions were utilised to clarify responses and explore participants' insights more deeply.

### Data analysis

Both workshops were audio-recorded, transcribed, and verified for accuracy. Using inductive thematic analysis, Author 2 conducted initial coding using NVIVO software, followed by Author 3’s review and further exploration. Findings were then shared with the team for additional discussion and refinement [14]. This process adhered to the Consolidated Criteria for Reporting Qualitative Research (COREQ) guidelines to ensure transparency and rigour [15].

### Ethics approval

Ethics approval for this project was obtained from Flinders University’s Human Research Ethics Committee (HREC) (Ref: #6149). Informed consent was obtained from all participants before data collection. Participants were informed about the potential use of their de-identified data for research and publication purposes.

## Results

**Fig. 2:**
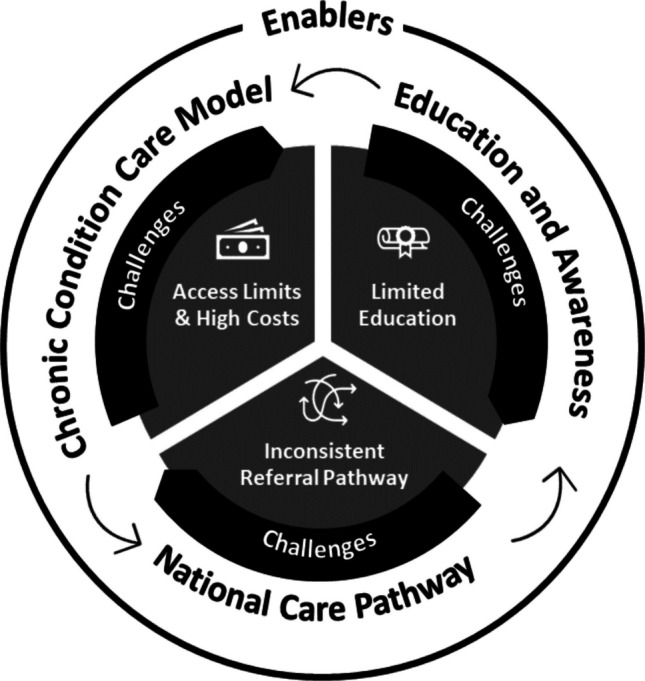
Conceptual framework illustrating the challenges and enablers of cancer-related lymphoedema care

Thematic analysis revealed two layers of findings. The first identified three key challenges in lymphoedema care management, whereas the second layer developed three practical solutions to address these challenges to provide direction for more consistent and patient-centred lymphoedema care management. Figure 2 summarises the study findings, highlighting the pillars for promoting consumer-centred, and multidisciplinary lymphoedema care.

Table 1 presents sample direct quotations from participants relevant to each theme.

**Table 1:** Sample direct quotes relevant to each theme

Themes	Direct quotes
1- Consumers’ key challenges	
Limited education and awareness of lymphoedema and its management	“I didn’t see the importance of lymphoedema—just not knowing anything about it—because I was just let go [after cancer treatment]” (Consumer) “As a 25-year-old girl, all of a sudden, I can’t put my high heels on, or I can’t wear a short skirt and feel pretty. So, it changed everything” (Consumer) “[…] the professional wrote a letter to my doctor who totally dismissed it. My oncologist totally dismissed it […], that’s a lack of education” (Consumer) “They told me it wouldn’t be aggravated by radiation, which, as we know now, it is” (Consumer)
Inconsistent and inequitable access to lymphoedema care, especially in regional and remote areas	“There is no one place to go; you have to try and figure it out on your own” (Consumer) “It’s almost like, I'm going to refer you to my friend John because I get a kickback. Not that they necessarily do, but that’s almost what it feels like […] they know them” (Consumer) “There’s referrals, but the referral pathway to clinics is very, very unclear… people are only being sent to a specific spot and then they’re being told that’s the only spot” (Consumer) “We’ve lost our lymphedema therapist in Bathurst, leaving many women without access to specialised care, which is frustrating” (Consumer) “It took me three years in Tassie to finally see a lymphoedema therapist, which was incredibly frustrating” (Consumer)
Financial burden of lymphoedema management	“Do I buy my children’s shoes, or do I get a garment?” (Consumer) “I go through a garment every three months. So, I’m going through eight stockings a year normally, and you get two covers. So, it’s still a big cost” (Consumer) “So paradoxically, if you have private health insurance, you actually are disadvantaged. And on top of that, I bet you that that information is not exactly easily available in terms of how to navigate it and how to maneuver the payment system […]” (Consumer) “I believe in some states there’s a rebate on power bills too, because of the heat, but in Queensland I don’t believe there is. And of all the states, it’s pretty warm here” (Consumer)
2- Enablers and proposed solutions for lymphoedema care	
Improve lymphoedema education for consumers and training for healthcare professionals	“My surgeon did believe in lymphoedema and actually handed me a sleeve and said, you’ll wear this from the day after surgery” (Consumer) “I manage it very well. For me, I’m very proactive with it. You will never see me without a garment” (Consumer) “We got an acting manager at the hospital who tried to stop early intervention, saying things had ‘blown out,’ but we were actually diagnosing people better and getting them seen quicker…They wanted to stop us being able to do early intervention… not paying for early intervention for people… I think it’s almost a sin, given that we know early intervention has its own set of benefits for many people” (Healthcare professional)
Holistic and coordinated care approach/care pathway	“Lymphoedema is important. It is also a very good example of a complex problem where you need to kind of harmonise solutions” (Healthcare professional) “[…] work out when to catch people and have a registry, so we know how many people are impacted by lymphoedema and from there, we go to the health economics” (Healthcare professional) “We need a registry […] the legacy of the registry as well […] looking at not just five-year data […], how we maintain and move forward into the next 100 years” (Healthcare professional)
Leveraging chronic condition models ○ Leveraging technology ○ Leveraging peer support	“What was mentioned before about lymphoedema as a chronic disease, so it should be incorporated into chronic disease management plans” (Healthcare professionals) “[…] tap into existing models like telehealth or virtual care clinics” (Healthcare professional) “I’ve learned more from these people than I ever have from my healthcare professionals because they also live with it every single day… […]” (Consumer) “My advocacy group, we lobbied, and we lobbied, so now Darwin has two [breast care nurses] … [] we now have a cancer care centre in Darwin” (Consumer)

### Key challenges

The following three themes were identified from the narratives of consumers and the experiences of healthcare professionals.

#### Limited education and awareness of lymphoedema and its management

Participants described feeling unprepared for the ongoing adjustments required to manage lymphoedema with an emphasis on self-care, employment, and social activities. Many identified that they were not informed about the potential for developing the condition and its management strategies early enough. The psychological burden of wearing compression garments, which served as visible reminders of their CRL, also impacted their self-image and emotional well-being. A lack of lymphoedema-specific education reported by healthcare professionals intensified these issues and was reported as contributing to inconsistent care, delayed diagnoses, and insufficient support for managing lymphoedema.

#### Inconsistent and inequitable access to lymphoedema care, especially in regional and remote areas

Many participants expressed frustration with lack of consistent referral pathways, particularly in rural and remote areas. Without clear guidelines and resources, consumers often had to navigate their own care independently, relying on the knowledge and networks of peers and local healthcare providers. This inconsistency created disparities in care, with some participants feeling that referrals were often based on convenience rather than clinical indications. The situation was worsened by limited access to lymphoedema care and services in regional or rural and remote areas, leaving many without the care they needed.

#### Financial burden of lymphoedema management

Participants highlighted the significant financial burden of managing lymphoedema, citing the high costs of essential compression garments and lifelong regular specialist appointments. These expenses often forced individuals to choose between managing their lymphoedema or attending to their basic needs. Government garment subsidies varied significantly across states, with some people identifying that annual subsidies were insufficient to meet their needs.

Even for those with private health insurance, the garment costs were described as prohibitive, with insurance coverage paradoxically limiting access to government support schemes. Navigating these payment systems in the context of managing CRL was often complex and overwhelming for participants.

### Enablers and proposed solutions for lymphoedema care

Building on the challenges identified, participants proposed three practical solutions to improve lymphoedema care management.

#### Improve lymphoedema education for consumers and training for healthcare professionals

Participants emphasised the need for comprehensive education for both cancer survivors and healthcare professionals, suggesting that a skilled workforce and informed consumers would foster a collaborative, supportive lymphoedema care ecosystem. Participants highlighted that trained healthcare professionals were crucial for early diagnosis, and engagement with proactive management strategies to prevent lymphoedema progression. Participants also felt that when they were well-informed about risks and management strategies, they gained confidence in managing their lymphoedema. This proactive approach could not only improve outcomes but also promote a more collaborative and supportive care environment.

#### Holistic and coordinated care approach/care pathway

Participants emphasised the importance of integrating CRL care into the broader cancer care model, prioritising a holistic approach that addresses physical, psychological, and social aspects through coordinated multidisciplinary teams and clear care pathway with navigation support. To address the challenges posed by the lack of a consistent care pathway, participants proposed establishing a national registry system. This registry would aim to address critical data gaps, particularly the lack of information on the prevalence of CRL, which currently makes the condition invisible to health policymakers. By providing comprehensive data on the number of individuals requiring CRL services, including garment subsidies, the registry could help build a strong case for consistent funding and resource allocation. Participants also viewed the registry as a tool to advocate for the development of consistent protocols, care pathways, and subsidies, ensuring equitable access to resources across regions.

#### Leveraging chronic condition models

Participants emphasised the importance of recognising lymphoedema as a chronic health condition requiring lifelong management strategies and aligning its management and funding models with other approaches to chronic disease management nationally. Participants emphasised opportunities in (i) leveraging technology and (ii) peer support that have been successfully deployed in other chronic conditions. Participants viewed digital health tools as bridging care gaps and promoting self-management. They also highlighted peer support as essential in the lymphoedema journey, providing emotional support and context-specific practical advice.

## Discussion

This study of stakeholder perspectives regarding CRL confirms the significant impact of CRL on daily life cancer survivors and significant gaps and challenges in lymphoedema care delivery as reported by both cancer survivors and healthcare professionals. These findings provide a unique Australian perspective of both healthcare professionals and cancer survivors across multiple cancer types that align with international literature [5, 16–18]. In addition, the participants provide recommendations on how to address the key challenges in the unique Australian context.

While no major differences emerged between the views of healthcare professionals and consumers, each group brought unique contributions to the discussion. This complementary dynamic underscores the alignment between consumer needs and professional insights, reinforcing the value of a collaborative approach to CRL care.

Consistent with broader literature [16, 19, 20], participants emphasised that improving education and awareness among healthcare professionals—including GPs, allied health professionals, and nurses—as well as individuals with or at risk of lymphoedema, is essential for effective management and better self-care practices [20, 21]. Greater knowledge and awareness support self-management of lymphoedema [16] and prevent the progression of lymphoedema symptoms and fosters a sense of ownership over their health, contributing to better overall outcomes [22, 23]. Additionally, enhanced awareness among healthcare professionals can help address the accessibility gaps in rural and regional areas, where the availability of specialists is often limited [24]. By equipping GPs, allied health professionals, nurses, and other relevant providers with the necessary knowledge and training, the availability of care in these regions can be improved. At the same time, empowering people through education and promoting early intervention that can be delivered in their primary care setting or through self-management can reduce the demand for specialised care, which is required for more advanced stages of lymphoedema, ensuring a more effective and efficient lymphoedema management approach across all areas.

A unique and novel contribution of this study was the emphasis on recognition of CRL as a chronic condition which should be managed in line with established frameworks for chronic disease management with emphasis on self-management and peer support [25, 26]. This approach aligns with the growing recognition that cancer and its symptoms benefit from chronic disease management approach, but it is complex, multidisciplinary, and dependent on consumer education and self-management support as well as clear care pathways and navigation support [27]. Peer support emerged as a key factor in enhancing lymphoedema self-management care [28] as digital health solutions in offering timely information and support [29–31]. These platforms could provide credible information, symptom monitoring tools, and enable communication with healthcare professionals and peers, promoting proactive disease management [29, 32, 33]. Furthermore, digital health interventions are cost-effective in managing chronic conditions through early intervention and awareness [34, 35]. Studies indicate that patient-centred care models with digital health lead to better outcomes, reduced costs, and improved treatment adherence [36].

The current absence of lymphoedema care pathway presents a challenge to navigation of fragmented services and reduced opportunities for early intervention [37]. This problem is further amplified by the fact that access and funding for services varies from state to state. Participants emphasised the importance of a single national approach to care consistent with National Optimal Care Pathways Framework [38] and supported by a national lymphoedema registry. Such a registry needs to be coupled with navigation services that monitor care needs and provide outreach to guide consumers and healthcare professionals in accessing resources, improving referral pathways, and facilitating connections to financial support.

This recommendation is particularly relevant as a recent AIHW report on lymphoedema burden in Australia identified lack of data is a major barrier to improvement of lymphoedema [4].

The challenges identified and solutions proposed in this study align completely with the goals and strategies outlined in the Australian Cancer Plan [39], which include focuses on improving health equity for people living rural and remote areas by developing innovative models of care including digital health and navigation; building the capability of the primary care workforce; improving access to optimal care; refining integrated care models to maximise evidence-based care; and maximising the leverage of data assets to support best possible care. As such, we propose to all Australian cancer care policy-makers and leaders that lymphoedema care and support should be used as a test case for implementing the Australian Cancer Plan.

## Limitations

This study has several limitations that should be acknowledged. Firstly, the participant sample was primarily drawn from individuals connected to existing networks, which may have introduced selection bias and limited the diversity of perspectives. Secondly, the study’s focus on the Australian healthcare system means that the identified challenges and proposed solutions may not be directly applicable to other countries with different healthcare structures. Lastly, as this research was exploratory and qualitative in nature, further quantitative studies are needed to validate the identified themes and assess the effectiveness of proposed interventions in larger, more diverse populations.

Future research should explore stakeholders’ perspectives leading to co-design and evaluation of interventions to improve lymphoedema care including digital tools for self-management support and navigation support and consideration for a national lymphoedema care pathway and registry.

## Conclusion

This study revealed significant challenges in managing cancer-related lymphoedema in Australia and proposed a range of practical solutions acceptable to cancer survivors and healthcare professionals alike to improve management of this complex condition.

Barriers to effective management included limited education and awareness, inconsistent and inequitable access to care, and the financial burden of management. To address these issues, participants proposed three practical solutions: improving education and training for consumers and healthcare professionals, establishing a holistic and coordinated care pathway, and aligning lymphoedema management with chronic disease models.

Advancing these solutions through a collaborative, person-centred approach could serve as a model that could be applied to non-cancer-related lymphoedema and other complex chronic conditions.

## Data Availability

The de-identified data we analysed are not publicly available, but requests to the corresponding author for the data will be considered on a case-by-case basis.

## References

[1] Bian J, Shen A, Yang W, Zhang L, Qiang W. Financial toxicity experienced by patients with breast cancer-related lymphedema: a systematic review. Support Care Cancer. 2023;31:354. 37237237 10.1007/s00520-023-07800-9

[2] Lentz R, Shin C, Bloom Z, Yamada K, Hong YK, Wong AK, et al. From bench to bedside: the role of a multidisciplinary approach to treating patients with lymphedema. Lymphat Res Biol. 2021;19:11–6. 33544026 10.1089/lrb.2020.0118 PMC8020565

[3] National Breast and Ovarian Cancer Centre. Review of research evidence on secondary lymphoedema: incidence, prevention, risk factors and treatment. Surry Hills (NSW): NBOCC; 2008 [cited 2024 Oct]. Available from: http://www.nbocc.org.au/breasthealth/careafter/lymphoedema.html.

[4] Australian Institute of Health and Welfare. 2024 Cancer data in Australia. Canberra: AIHW; 2024. https://www.aihw.gov.au/reports/cancer/cancer-data-in-australia/data (Viewed Oct 2024).

[5] Burckhardt M, Belzner M, Berg A, Fleischer S. Living with breast cancer-related lymphedema: a synthesis of qualitative research. Oncol Nurs Forum. 2014;41:E220-237. 24969257 10.1188/14.ONF.E220-E237

[6] DiSipio T, Rye S, Newman B, Hayes S. Incidence of unilateral arm lymphoedema after breast cancer: a systematic review and meta-analysis. Lancet Oncol. 2013;14:500–15. 23540561 10.1016/S1470-2045(13)70076-7

[7] Fu MR, Chen CM, Haber J, Guth AA, Axelrod D. The effect of providing information about lymphedema on the cognitive and symptom outcomes of breast cancer survivors. Ann Surg Oncol. 2010;17:1847–53. 20140528 10.1245/s10434-010-0941-3

[8] Moffatt CJ, Franks PJ, Doherty DC, Williams AF, Badger C, Jeffs E, et al. Lymphoedema: an underestimated health problem. QJM. 2003;96:731–738. 10.1093/qjmed/hcg126 14500859

[9] Lawn S, Fallon-Ferguson J, Koczwara B. Shared care involving cancer specialists and primary care providers – what do cancer survivors want? Health Expect. 2017;20:1081–7. 28467626 10.1111/hex.12551 PMC5600229

[10] Sierla R, Black D, Lee TS, Kilbreath S. Access to treatment for breast cancer-related lymphoedema in Australia. Aust Fam Physician. 2013;42:892–5. 24324994

[11] Torgbenu E, Luckett T, Buhagiar M, Requena CM, Phillips JL. Improving care for cancer-related and other forms of lymphoedema in low- and middle-income countries: a qualitative study. BMC Health Serv Res. 2022;22:461. 35395942 10.1186/s12913-022-07840-7 PMC8990607

[12] Omidi Z, Kheirkhah M, Abolghasemi J, Haghighat S. Effect of lymphedema self-management group-based education compared with social network-based education on quality of life and fear of cancer recurrence in women with breast cancer: a randomized controlled clinical trial. Qual Life Res. 2020;29:1789–800. 32152817 10.1007/s11136-020-02455-z PMC7295820

[13] Kruger N, Plinsinga ML, Noble-Jones R, Piller N, Keeley V, Hayes SC. The lymphatic system, lymphoedema, and medical curricula-survey of Australian medical graduates. Cancers (Basel). 2022;14:6219. 36551705 10.3390/cancers14246219 PMC9777454

[14] Braun V, Clarke V. Thematic analysis: a practical guide. London: SAGE Publications; 2021.

[15] Tong A, Sainsbury P, Craig J. Consolidated criteria for reporting qualitative research (COREQ): a 32-item checklist for interviews and focus groups. Int J Qual Health Care. 2007;19:349–57. 17872937 10.1093/intqhc/mzm042

[16] Jeffs E, Ream E, Shewbridge A, Cowan-Dickie S, Crawshaw D, Huit M, et al. Exploring patient perception of success and benefit in self-management of breast cancer-related arm lymphoedema. Eur J Oncol Nurs. 2016;20:173–83. 26338435 10.1016/j.ejon.2015.08.001

[17] International Society of Lymphology. The diagnosis and treatment of peripheral lymphedema: consensus document of the International Society of Lymphology. Lymphology. 2013 [cited 2024 Nov];46(1):1–11. Available from: https://pubmed.ncbi.nlm.nih.gov/12926833/. 23930436

[18] Río-González Á, Molina-Rueda F, Palacios-Ceña D, Alguacil-Diego IM. Comparing the experience of individuals with primary and secondary lymphoedema: a qualitative study. Braz J Phys Ther. 2021;25:203–13. 32518025 10.1016/j.bjpt.2020.05.009 PMC7990727

[19] Jeffs E, Wiseman T. Randomised controlled trial to determine the benefit of daily home-based exercise in addition to self-care in the management of breast cancer-related lymphoedema: a feasibility study. Support Care Cancer. 2013;21:1013–23. 23073712 10.1007/s00520-012-1621-6

[20] Ridner SH, Dietrich MS, Kidd N. Breast cancer treatment-related lymphedema self-care: education, practices, symptoms, and quality of life. Support Care Cancer. 2011;19:631–7. 20393753 10.1007/s00520-010-0870-5

[21] Cansız G, ArıkanDönmez A, Kapucu S, Borman P. The effect of a self-management lymphedema education program on lymphedema, lymphedema-related symptoms, patient compliance, daily living activities and patient activation in patients with breast cancer-related lymphedema: a quasi-experimental study. Eur J Oncol Nurs. 2022;56:102081. 34875398 10.1016/j.ejon.2021.102081

[22] Radina ME, Armer JM, Stewart BR. Making self-care a priority for women at risk of breast cancer–related lymphedema. J Fam Nurs. 2014;20:226–49. 24476674 10.1177/1074840714520716

[23] Zhao H, Wu Y, Zhou C, Li W, Li X, Chen L. Breast cancer-related lymphedema patient and healthcare professional experiences in lymphedema self-management: a qualitative study. Support Care Cancer. 2021;29:8027–44. 34226959 10.1007/s00520-021-06390-8

[24] Schulze H, Nacke M, Gutenbrunner C, Hadamitzky C. Worldwide assessment of healthcare personnel dealing with lymphoedema. Health Econ Rev. 2018;8:10. 29663122 10.1186/s13561-018-0194-6 PMC5901432

[25] Venchiarutti RL, Dhillon H, Ee C, Hart NH, Jefford M, Koczwara B. Priorities for multimorbidity management and research in cancer: a Delphi study of Australian cancer survivors, clinicians, and researchers. J Cancer Surviv. 2024. 10.1007/s11764-024-01686-0. Epub ahead of print. Available from: https://pubmed.ncbi.nlm.nih.gov/39354281/. 10.1007/s11764-024-01686-0 PMC12988988 39354281

[26] Australian Health Minister s’ Advisory Council. 2020 National strategic framework for chronic conditions. Canberra: Australian Government; 2020. https://www.health.gov.au/sites/default/files/documents/2019/09/national-strategic-framework-for-chronic-conditions.pdf (Viewed Oct 2024).

[27] Pituskin E. Cancer as a new chronic disease: oncology nursing in the 21st century. Can Oncol Nurs J. 2022;32:87–92. 35280062 PMC8849169

[28] Thompson DM, Booth L, Moore D, Mathers J. Peer support for people with chronic conditions: a systematic review of reviews. BMC Health Serv Res. 2022;22:427. 35361215 10.1186/s12913-022-07816-7 PMC8973527

[29] Buckingham SA, Williams AJ, Morrissey K, Price L, Harrison J. Mobile health interventions to promote physical activity and reduce sedentary behaviour in the workplace: a systematic review. Digit Health. 2019;5:1–50. 10.1177/2055207619839883 PMC6437332 30944728

[30] Fan K, Zhao Y. Mobile health technology: a novel tool in chronic disease management. Intell Med. 2022;2:41–7.

[31] Hemati M, Rivaz M, Khademian Z. Lymphedema self-management mobile application with nurse support for post breast cancer surgery survivors: description of the design process and prototype evaluation. BMC Cancer. 2024;24:973. 39118042 10.1186/s12885-024-12744-2 PMC11308577

[32] Jourdain P, Pages N, Amara W, Maribas P, Lafitte S, Lemieux H, et al. Perceptions and satisfaction of patients with chronic heart failure when using a remote monitoring web application named Satelia® Cardio. Ann Cardiol Angeiol (Paris). 2023;72:101606. 37244215 10.1016/j.ancard.2023.101606

[33] Law L, Kelly JT, Savill H, Wallen MP, Hickman IJ, Erku D, et al. Cost-effectiveness of telehealth-delivered diet and exercise interventions: a systematic review. J Telemed Telecare. 2022;30:420–37. 35108135 10.1177/1357633X211070721

[34] Fatoye F, Gebrye T, Mbada C, Useh U. Economic evaluations of digital health interventions for the management of musculoskeletal disorders: systematic review and meta-analysis. J Med Internet Res. 2023;25:e41113. 37410542 10.2196/41113 PMC10359913

[35] Zangger G, Bricca A, Liaghat B, Juhl CB, Mortensen SR, Andersen RM, et al. Benefits and harms of digital health interventions promoting physical activity in people with chronic conditions: systematic review and meta-analysis. J Med Internet Res. 2023;25:e46439. 37410534 10.2196/46439 PMC10359919

[36] Schofield P, Shaw T, Pascoe M. Toward comprehensive patient-centric care by integrating digital health technology with direct clinical contact in Australia. J Med Internet Res. 2019;21:e12382. 31165713 10.2196/12382 PMC6682300

[37] Stout NL, Binkley JM, Schmitz KH, Andrews K, Hayes SC, Campbell KL, et al. A prospective surveillance model for rehabilitation for women with breast cancer. Cancer. 2012;118(8 Suppl):2191–200. 22488693 10.1002/cncr.27476

[38] Cancer Australia. 2024 national optimal care pathways framework. New South Wales (NSW): Australian Government; 2024 [cited 2024 Nov]. Available from: https://www.canceraustralia.gov.au/sites/default/files/2025-01/national-optimal-care-pathways-framework.pdf.

[39] Cancer Australia. 2023 Australian cancer plan. New South Wales (NSW): Australian Government; 2023 [cited 2024 Nov]. Available from: https://www.canceraustralia.gov.au/sites/default/files/publications/pdf/2023_ACP%20Summary%20Report%20DIGITAL_V9.pdf.

